# Supplementary figures and images for: Optimized phylogenetic clustering of HIV-1 sequence data for public health applications
Source: PLoS Comput Biol. 2022 Nov 30;18(11):e1010745. doi: 10.1371/journal.pcbi.1010745 (PMC9744331; doi:10.1371/journal.pcbi.1010745)

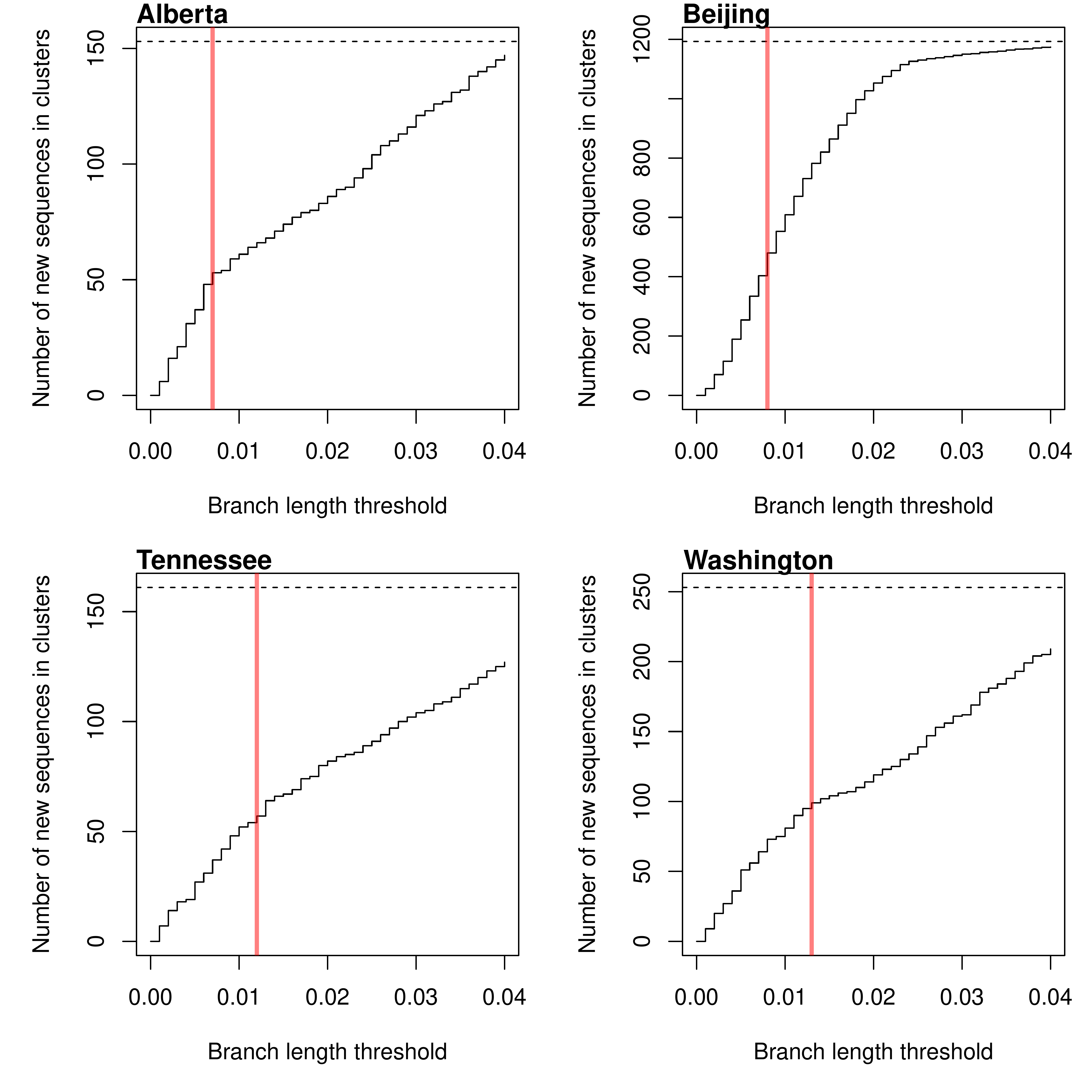

Supplement: S1 Fig — The number of new cases that join any cluster (ie. the total cluster growth) plotted against the branch length threshold used to define clusters. The optimal threshold determined in Fig 4 for each data set is marked in red. (TIF) [file pcbi.1010745.s001.tif]

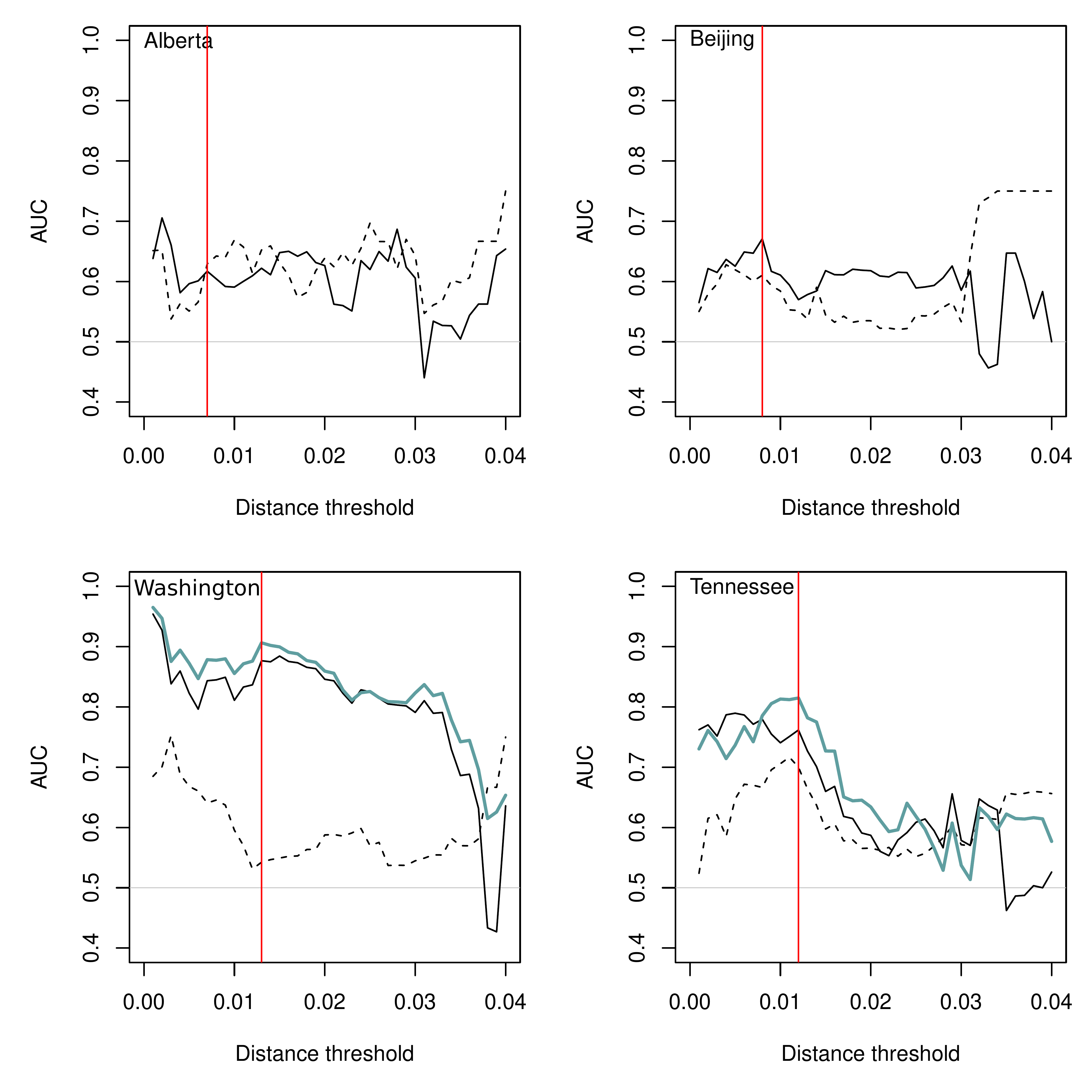

Supplement: S2 Fig — The AUC results for receiver operator characteristic (ROC) curves for the prediction of whether or not a cluster would acquire new cases based on recency (collection date) plotted against the distance threshold used to define clustering. The same result was also calculated without a bootstrap requirement for clustering (dashed) and with diagnostic dates used to measure recency (green). The optimal threshold determined in Fig 4 for each data set is marked in red. (TIF) [file pcbi.1010745.s002.tif]

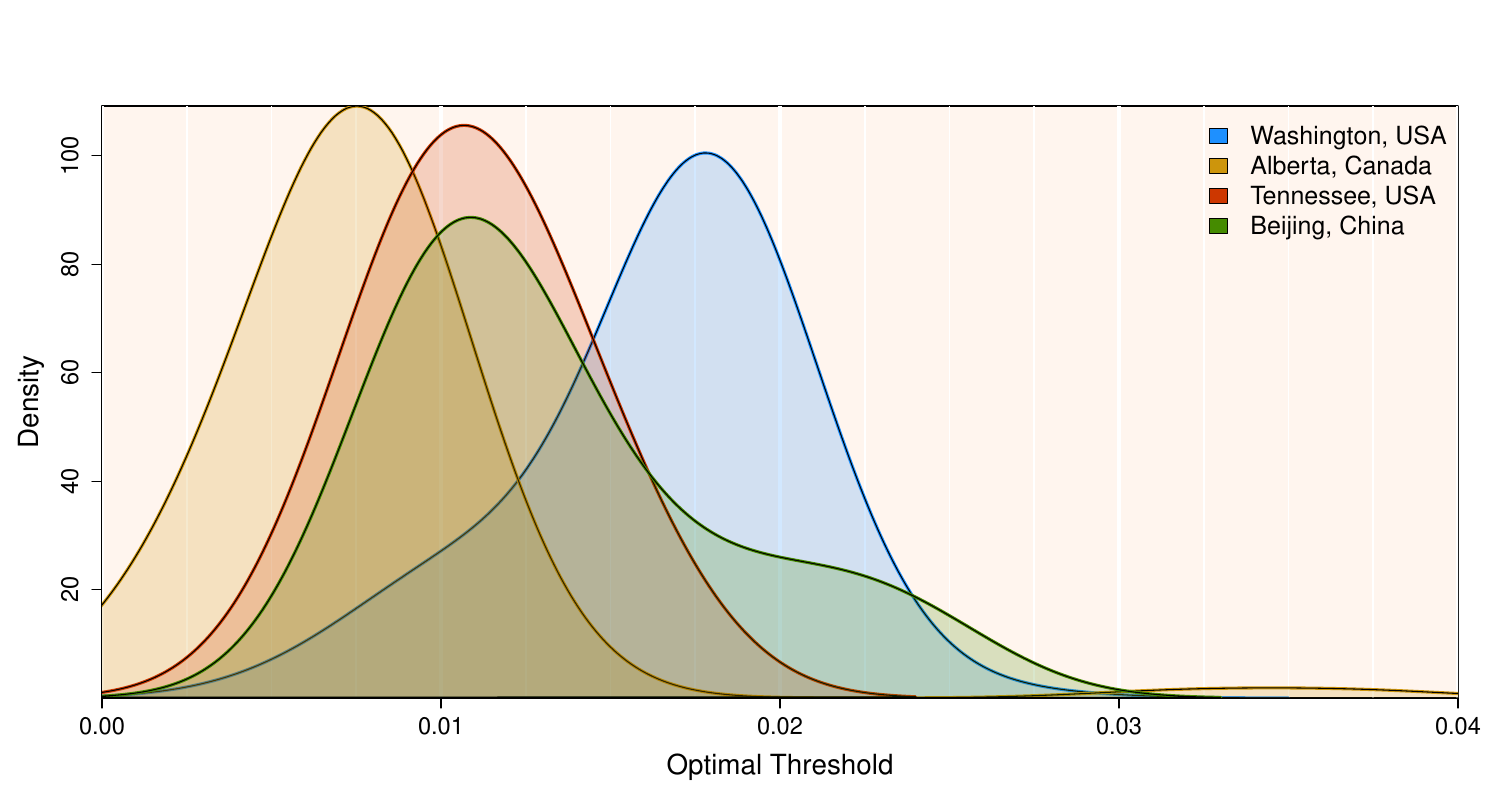

Supplement: S3 Fig — The distribution of maximum branch length thresholds which resulted in the largest difference in AIC between Poisson-linked models of cluster growth for 100 approximate likelihood trees built on 80% subsamples of the old sequences without replacement. Clusters and growth are defined at 41 different maximum distance thresholds from 0 to 0.04 with a minimum bootstrap support requirement of 95% for ancestral nodes. The AIC of a null model where size predicts growth is subtracted from the AIC of a proposed model where size and mean time predict growth. (TIF) [file pcbi.1010745.s003.tif]

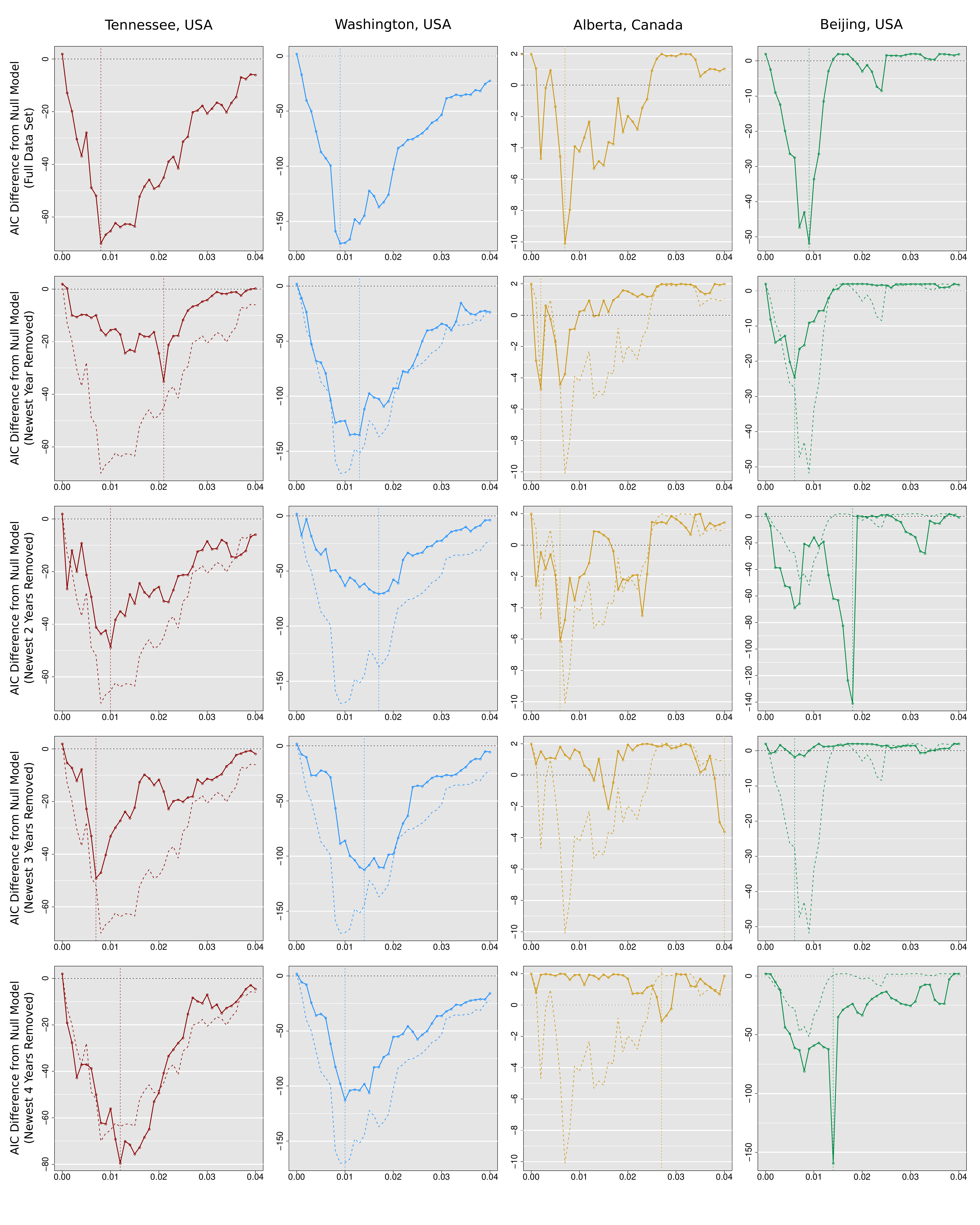

Supplement: S4 Fig — Difference in AIC (ΔAIC) between Poisson-linked models of cluster growth for four separate data sets (Tennessee, USA; Washington, USA; Alberta, Canada; Beijing, China). Clusters and growth are defined at 41 different maximum distance thresholds from 0 to 0.04. The AIC of a null model where size predicts growth is subtracted from the AIC of a proposed model where size and mean time (diagnostic date for USA Data, collection dates for others) predict growth. The top row of plots used the complete data sets and each following row excludes an additional year of sample collection. ΔAIC profiles for the complete data are displayed as a dashed line on each subsequent plot for reference. The minimum value in each plot is marked with a vertical line. (TIF) [file pcbi.1010745.s004.tif]

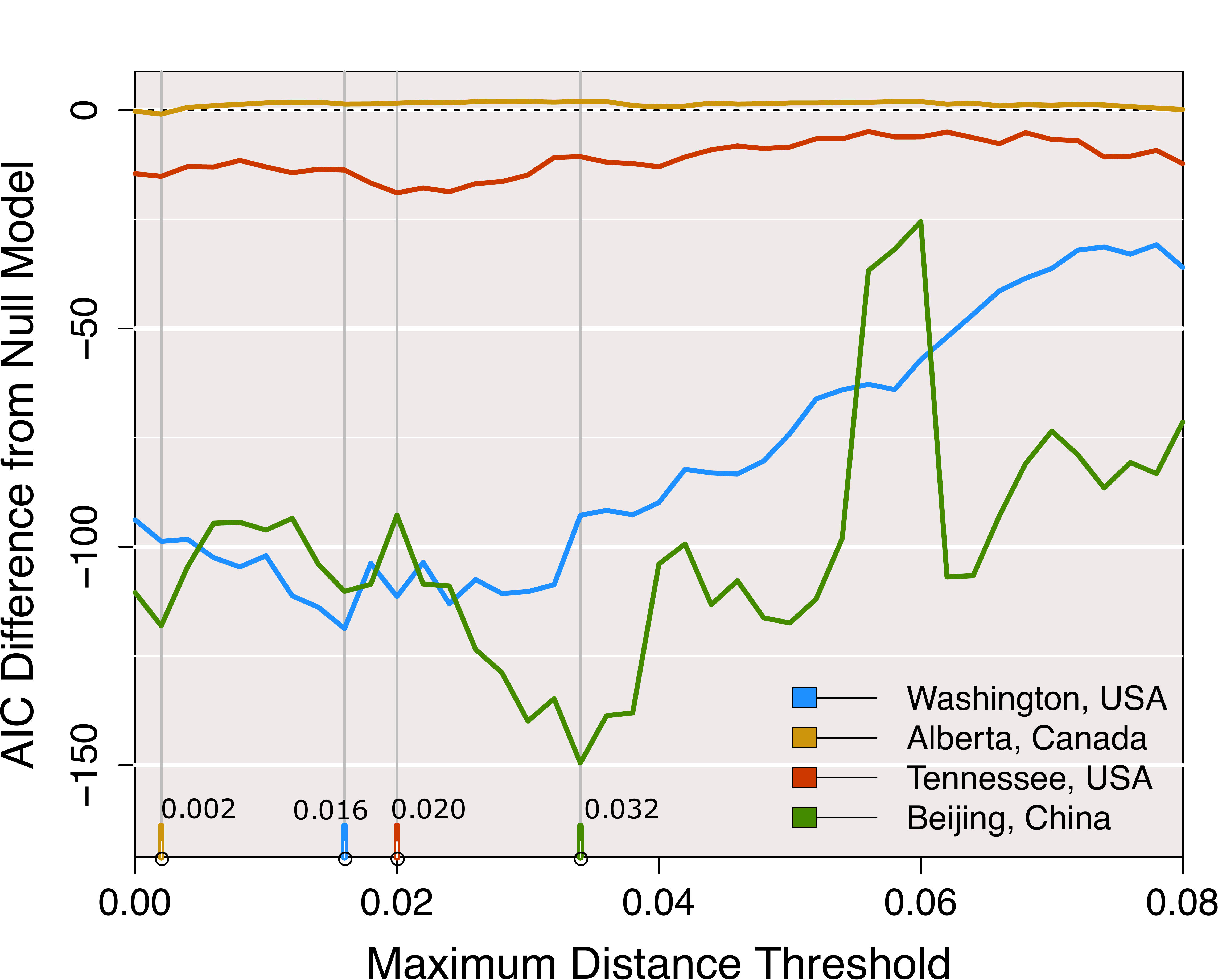

Supplement: S5 Fig — Each line represents the difference in AIC between Poisson-linked models of cluster growth for different data sets (see inset legend). Clusters and growth are defined at 41 different maximum patristic distance thresholds within monophyletic clades from 0 to 0.04 with a minimum bootstrap support requirement of 95% for ancestral nodes. The AIC of a null model where size predicts growth is subtracted from the AIC of a proposed model where size and mean time predict growth. (TIF) [file pcbi.1010745.s005.tif]

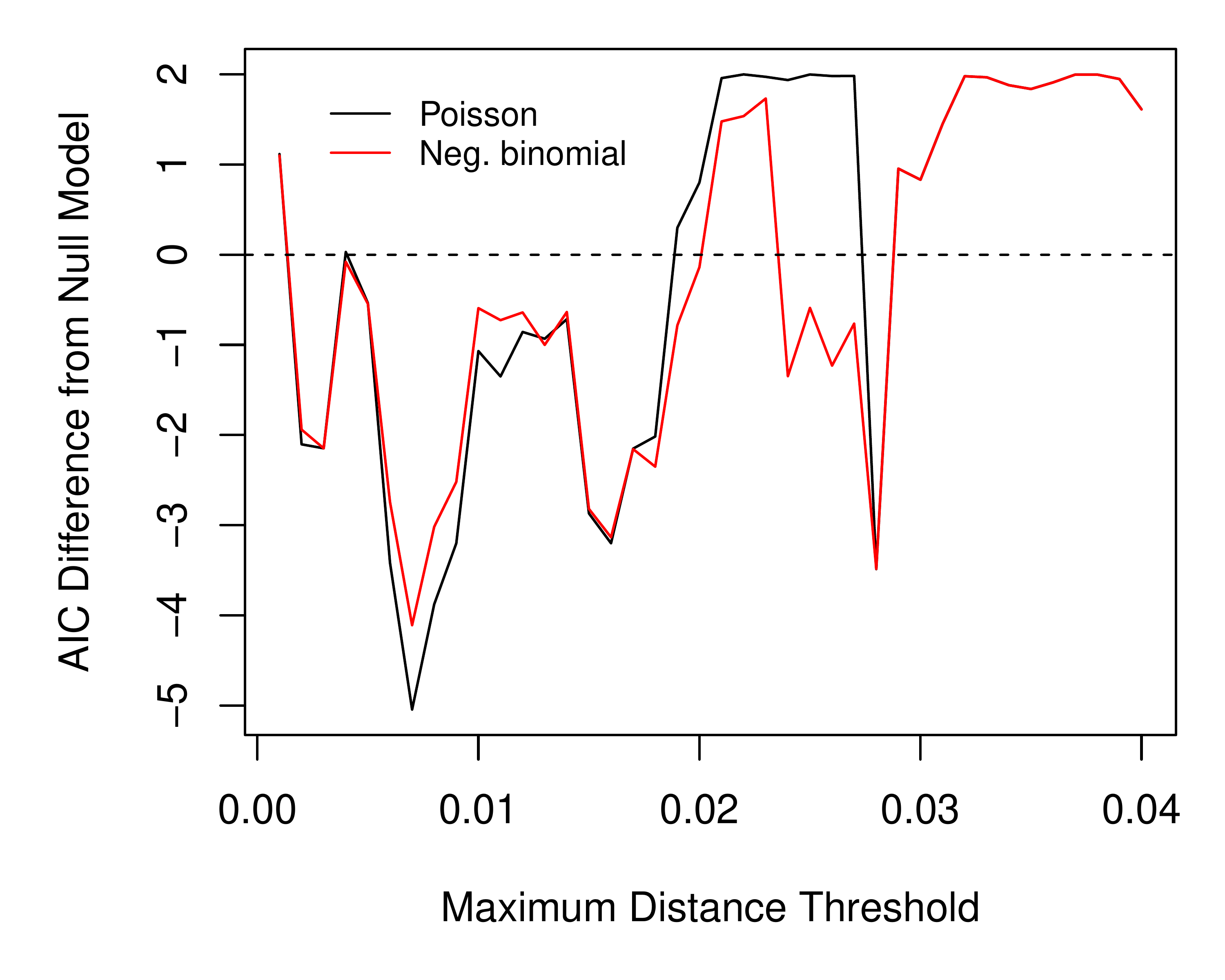

Supplement: S6 Fig — Difference in AIC between null and alternative models using Poisson (black) and negative binomial (red) regressions on cluster growth for the middle Tennessee data set. We used the glm.nb function in the R package MASS [84] to fit negative binomial regression models. Clusters and growth are defined at 40 different maximum patristic distance thresholds within monophyletic clades from 0.001 to 0.04 with a minimum bootstrap support requirement of 95% for ancestral nodes. The AIC of a null model where size predicts growth is subtracted from the AIC of a proposed model where size and mean time predict growth. (TIF) [file pcbi.1010745.s006.tif]
