# Supplementary material for: Optimized phylogenetic clustering of HIV-1 sequence data for public health applications
Source: PLoS Comput Biol. 2022 Nov 30;18(11):e1010745. doi: 10.1371/journal.pcbi.1010745 (PMC9744331; doi:10.1371/journal.pcbi.1010745)
Supplement: Attachment — Submitted filename: cover-letter-2.pdf [file pcbi.1010745.s007.pdf]

Schulich
MEDICINE & DENTISTRY

Pathology and
Laboratory Medicine

July 12, 2022

Dear Editors of *PLOS Computational Biology*,

On behalf of my co-authors, we are submitting a revised version of 'Optimized phylogenetic clustering of HIV-1 sequence data for public health applications' for your consideration. We are grateful to the reviewers and editors for their thoughtful evaluation of the original manuscript, and for the opportunity to address their comments with a revised version.

Below, we have itemized the comments from the editor and reviewers, each accompanied by a response explaining how our revisions address the comment with reference to the manuscript.

Editor

1. *"Can the authors provide data on the trees used for these studies and/or more specific information on how to request access to the data? In addition, can you also make available all scripts used to run the analysis. In its current form, I do not believe the work is reproducible even if one were to receive access to the raw data. Addressing data availability concerns will be a criteria for publication."*

   We have released all the scripts used to run the cluster optimization analysis in a public GitHub repository at https://github.com/PoonLab/tn.

   We agree that providing data on the trees is an important step for enabling reviewers and readers to reproduce our analysis. The analysis workflow that we develop in our paper requires both a multiple alignment of genetic sequences and a maximum likelihood reconstruction of a tree relating these sequences. (Sequences are required because those representing new infections are 'grafted' onto the tree by maximum likelihood.) One of the data sets (from northern Alberta) was deposited by the original authors in GenBank, and we have made our alignment and tree for those data available at the above URL.

   Due to the highly sensitive nature of HIV-1 sequence data, we are working under severe restrictions on data sharing. However, we have reached an agreement with our collaborators to provide the remaining three data sets as re-anonymized, randomized sequence alignments that are sufficient to reproduce our analyses. Each alignment was generated by applying a random permutation to the columns of the original alignment, resulting in sequences that bear no resemblance to HIV-1 while retaining their phylogenetic relationships. We also replaced the sequence labels with arbitrary indices and reduced the precision of sample collection dates to years, which was the time unit used for our analysis. These data are now available at the above URL. Finally, we now provide contact information for readers to submit data access requests to authorized groups or individuals from the respective laboratories.

   We have updated our *Data and code availability* statement to reflect these changes.

2. *“ Given the sampling scheme? Why isn’t a time-calibrated tree in BEAST more appropriate? I am not requiring the authors to make this change, but asking for–as a minimum–clarification in the text. ”*

   Sampling time-calibrated trees from the posterior distribution in BEAST enables users to incorporate prior information and accommodate complex model uncertainty. However, Bayesian sampling methods are generally limited to trees relating no more than about 500 sequences, due to the challenge of attaining model convergence with an enormous number of possible trees. Since public health applications of phylogenetic clustering for HIV-1 can involve tens of thousands of sequences, it is usually not feasible to adopt a Bayesian approach to this problem. Some studies have attempted to overcome this limitation by applying BEAST to smaller subtrees extracted from a larger maximum likelihood tree. However, Dearlove, Xiang and Frost (2017) previously determined that this approach induces biased phylogenetic clustering.

   We have modified the Discussion section of our manuscript to identify Bayesian sampling as a potential area for further method development.

3. *“Is there a concern about over-dispersion and have to tried fitting a negative binomial model? In my experience, this often doesn’t matter, so it’s ok to simply say as much in the discussion.”*

   We verified that using the negative binomial has only a slight effect on the differences in AIC between models (now added as Supporting Information Figure S6). We have also included the investigation of overdispersion in the number of incident cases among clusters as a direction for further work in the Discussion.

4. *“I was surprised to see the code licensed under GPL v3 instead of MIT. Was there a reason for this decision?”*

   The GNU General Public License (GPL) is frequently chosen by open-source developers for releasing bioinformatic programs, such as MAFFT and IQ-TREE. For instance, there are 67 mentions of the GPL in the journal *PLOS Computational Biology*, compared to 38 mentions of the MIT license. In addition, we are obligated to release our source code under the terms of the GPL because it includes binaries for the program *pplacer*, which was released under this license.

   The code that we released at https://github.com/PoonLab/clustuneR represents an extensive refactoring of our project code to make it more user friendly. However, we decided to release the original project source code under the same license at https://github.com/PoonLab/tn to make the study methods as reproducible as possible.

Reviewer 1

1. *“The concept of grafting new sequences into a tree is interesting and innovative, particularly for public. A comparison with a more traditional approach used currently in surveillance of molecular HIV clusters would be helpful and could serve, as a further validation.”*

Thank you for raising this point. Making such a comparison was the objective of our section on monophyletic clustering, which is a common approach to phylogenetic clustering (consider, for example, ClusterPicker). We have modified the text to clarify this. In addition, we have expanded on the comparisons between our current work and previous work with pairwise distance clustering, which has 'traditionally' been used for the molecular surveillance of clusters.

2. *"Some topics in Introduction, like, SARS-CoV-2, are not directly related to the study and could be omitted or shortened. "*

   We have greatly reduced mention of the SARS-CoV-2 pandemic in the revised manuscript.

3. *"Discussion includes a thorough literature review, which rather belongs to Introduction."*

   We have relocated some of our review of the literature to the Introduction section, and revised both sections to adjust for this change.

4. *"The study limitations might need to be presented and discussed. "*

   We have expanded on the limitations of our method in the Discussion section, such as overdispersion in the number of new cases among known cases (*i.e.*, the negative binomial), and relying on a single point estimate of the tree (maximum likelihood versus Bayesian methods).

5. *"Full and short titles are identical."*

   We modified the short title to 'Optimized phylogenetic clustering'.

6. *"Figure 1 legend: The last sentence does not specify that it refers to Figure 1E; needs update."*

   We have made this correction, thank you.

Reviewer 2

1. *"The authors have performed sensitivity analyses whereby they randomly reduced the amount of background sequences to 80%. However, a more critical sensitivity analyses, in my opinion, is how the inferred optimal distance threshold for each geographic location would change with time. The authors currently used the most recent year of sequence collected for each of the four datasets as the 'incident' subset of sequences. Can the authors perform the same analyses for a sample of historical snapshots in the past? For instance, for the Washington dataset, the authors have currently used all sequences collected before 2019 as the "background" set while 2019, the most recent year as the "incident" set. What about if the "most recent year" is now 2010 while the "background" set constitutes all data collected before 2010? How much does the distance threshold change with time in different timescales (e.g. compare between 2010 and 2019 vs between 2018 and 2019)? How much of this change then has to do with actual meaningful known difference in transmission patterns and how much of it is due simply because of variations in the amount of sequences available?"*

We agree with the reviewer that it would be useful to determine the sensitivity of our method to differences in sampling timescales. Thus, we evaluated the extent that the optimal distance threshold varied at different timescales by progressively right-censoring the Tennessee and Seattle data sets (Figure S3). We observed that the optimal thresholds varied measurably over time. For example, the optimal distance for the Seattle data set varied from 0.009 to 0.017 with different censoring time points from 2018 to 2014. This range of thresholds was comparable to what we observed for random sub-sampling of 80% of the data. These results have been incorporated into the revised manuscript.

2. *"While I largely agree with the proposed framework that is fundamentally using sampling dates to systematically aid transmission cluster definition, the manuscript in its current form does not explicitly present any evaluation of how the distance thresholds inferred through this framework improve the interpretation and accuracy of the identified transmission clusters to known epidemiologically confirmed/likely transmission networks."*

   We apologize for this misunderstanding. It is not our objective to reconstruct direct person-to-person transmission events. Several studies have indeed attempted this through the comparative analysis of HIV-1 sequences, followed by validation against 'known' epidemiologically confirmed or likely transmission networks. This reconstruction of transmission events at the level of individuals has both epidemiological and forensic applications, along with significant ethical issues. In contrast, our method is designed to provide a means of prioritizing clusters for public health measures by optimizing the prediction of the *number* of new infections per cluster. Whether or not the genetic similarity between infections represents a direct transmission event between individuals is irrelevant.

   To avoid further misunderstanding, we have added to the Discussion section to clarify the purpose of our work.

3. *"SARS-CoV-2 transmission routes and timescales, evolutionary dynamics as well as its epidemiology are drastically distinct from HIV-1. Often times, it is difficult to identify clear transmission clusters using phylogenetic methods for SARS-CoV-2. Unless the authors are able to show clear utility of the method for identifying SARS-CoV-2 transmission clusters, I find the last sentence in line 450 misleading. "*

   We have removed this sentence from the revised manuscript.

Sincerely,

Art F. Y. Poon, PhD

Department of Pathology and Laboratory Medicine
Schulich School of Medicine & Dentistry, Western University
Health Sciences Addition, Room H422, London ON, Canada N6A 5C1
t: 519-661-2111 ext. 87978   e: apoon42@uwo.ca

Western
