# Supplementary material for: Optimized phylogenetic clustering of HIV-1 sequence data for public health applications
Source: PLoS Comput Biol. 2022 Nov 30;18(11):e1010745. doi: 10.1371/journal.pcbi.1010745 (PMC9744331; doi:10.1371/journal.pcbi.1010745)
Supplement: Attachment — Submitted filename: cover-letter-3.pdf [file pcbi.1010745.s008.pdf]

Schulich
MEDICINE & DENTISTRY

Pathology and
Laboratory Medicine

October 11, 2022

Dear Editors of *PLOS Computational Biology*,

On behalf of my co-authors, I thank the editor and reviewers for their time in re-assessing our revised manuscript, and for the opportunity to make additional improvements in response to their feedback. Below, we have itemized the remaining comments from the second reviewer, each accompanied by a response explaining how our revisions address the comment with reference to the manuscript.

Reviewer #2

1. *"Why are only the Tennessee and Washington datasets used for the right-censoring sensitivity analyses, while the Alberta and Beijing datasets were excluded? The authors have sequence data collected over 7-10 years for Alberta and Beijing as well. In particular for Beijing, the authors observed a much broader ΔAIC curve in the 80% subsampling sensitivity analyses and that was suggested to be due to atypically higher incidence. Did they observe a similar broad curve on a year with greater incidence? If so, isn't rapid high incidence a limitation that affects the sensitivity of this framework? "*

   We agree with the reviewer that the right-censoring sensitivity analysis should include the Alberta and Beijing data sets. We had anticipated that right-censoring would be problematic for these data sets, due to lower overall sample size (Alberta), or substantial variation in sample sizes over time (Beijing). Results including Alberta and Beijing have been incorporated as an expanded version of Supporting Information (SI) Figure 3. As expected, the location of ΔAIC optimized distance thresholds is very unstable over time for the Alberta and Beijing data sets, which we now emphasize in the Results section as a limitation of the method.

2. *" I am quite confused by the authors' rationalisation of my second critique. I agree that there are ethical issues surrounding the forensic use of sequence data and phylogenetic analyses to identify HIV-1 transmission events. However, the debate surrounding this issue centres on, in my opinion and I believe the authors', the unjust criminalisation of HIV transmissions and the ensuing demonstrable negative impacts they have on HIV public health in certain countries/communities. I disagree with the authors' reasoning that because such data and analyses may be used to prosecute HIV infected individuals, that exempts them from validating if the clusters they had identified using their framework are accurately linked epidemiologically. Even if the authors' framework is "designed to provide a means of prioritising clusters for public health measures by optimising the prediction of the number of new infections per cluster", there is a need to know if the prioritised clusters are in fact accurately linked epidemiologically, and if the grafted sequences (the prospective cases) are in fact correctly placed to known clusters. This is especially important if the authors claim that their method is superior over other currently-available methods in determining the optimal*

clustering criteria for public health applications. "

We apologize that we misunderstood the reviewer's previous comment, and we are glad that we share the same views on the ethical issues surrounding forensic applications of sequence analysis to reconstruct HIV-1 transmission events. To address the reviewer's request to evaluate whether "grafted sequences [...] are in fact correctly placed to known clusters", we carried out a simulation-based validation experiment. In brief, we used the program FAVITES to generate a contact network among 26,746 individuals under a preferential attachment model, and then to simulate the ten-year spread of an epidemic through this network using a compartmental model that was calibrated to HIV-1 transmission dynamics, including effects of acute infections and treatment on transmission rates. We applied our method to the resulting set of HIV-1 sequences and evaluated the accuracy of grafting sequences to the correct clusters.

Overall, we found that when the true transmission source associated with an incident sequence had been sampled as sequence in the data set, our method correctly placed new sequences into a cluster containing that source about 99.5% of the time for no bootstrap threshold, and 88.5% of the time for a bootstrap threshold of 95%, when using ΔAIC-optimized distance thresholds. If the true source individual had *not* been sampled, then we determined the shortest distance (in edges) in the true transmission tree to any member of the phylogenetic cluster. These new results are presented in Figure 5 of the revised manuscript, along with substantial additions to the Methods and Results section.

3. *"Please be consistent with your reference to the Washington dataset throughout the text - either stick with "Washington" or "Seattle". "*

   Thank you for pointing this out. We have switched to using 'Washington' consistently throughout the manuscript.

4. *"Could you please plot the ΔAIC curve for the full tree against that inferred for each right-censored data in Figure S3 like what you did in Figure 3?"*

   We have added the ΔAIC profiles for the full tree for each data set in Figure 3 as dashed lines in Figure S3.

Sincerely,

Art F. Y. Poon, PhD

Department of Pathology and Laboratory Medicine
Schulich School of Medicine & Dentistry, Western University
Health Sciences Addition, Room H422, London ON, Canada N6A 5C1
t: 519-661-2111 ext. 87978 e: apoon42@uwo.ca

Western
